# Physical Diagnosis and Practical Urinalysis

**Published:** 1890-11

**Authors:** 


PHYSICAL DIAGNOSIS AND PRACTICAL URINALYSIS. An Epitome of the Physical Signs of the Heart, Lung, Kidney, and Spleen in Health and Disease. Edited by John E. Clark, M. D., Professor of General Chemistry and Physics in the Detroit College of Medicine. Forty-one illustrations. Cloth, 12mo, two hundred pages; price, postpaid, $1.00. Illustrated Medical Journal Co., Publishers, Detroit, Mich.

In the arrangement of this work the object has been to present to the medical student and practitioner a systematic and condensed course of Physical Diagnosis and Urinalysis. The portion on Urinalysis will be found to consist of two parts, practical and reference. The editor believes there is a demand, in many medical schools and by many medical students, for a short, definite course of organic chemistry, touching alone on those subjects of everyday interest to the medical practitioner, such as the analysis of urine, chemical and microscopical; the examination of sputa, bile, blood, bacteria, etc.; methods for the quantitative estimation of the more important urinary constituents, normal and abnormal, such as urea, chlorides, sugar, albumen, etc. To meet these requirements the editor has compiled this volume. Teachers in the laboratory will find the work of advantage as giving the plan for definite instruction with such manipulatory details as will enable students to pursue the course of urine analysis with the minimum of assistance. This is essentially the same as the course given by the editor in the college with which he is connected. Plates have been introduced as needed to still further assist in elucidating the text.

